# Acute Physiological and Psychological Stress Response in Youth at Clinical High-Risk for Psychosis

**DOI:** 10.3389/fpsyt.2021.641762

**Published:** 2021-02-19

**Authors:** Emily E. Carol, Robert L. Spencer, Vijay A. Mittal

**Affiliations:** ^1^Laboratory for Early Psychosis (LEAP) Center, Psychotic Disorders Division, McLean Hospital and Harvard Medical School, Belmont, MA, United States; ^2^Spener Neuroendocrinology Laboratory, Department of Psychology and Neuroscience, University of Colorado Boulder, Boulder, CO, United States; ^3^Adolescent Development and Preventative Treatment Program, Departments of Psychology, Psychiatry, and Medical Social Sciences, Institute for Policy Research, Northwestern University, Evanston, IL, United States

**Keywords:** psychosis, high-risk, stress reactivity, HPA-axis, alpha amylase, cortisol

## Abstract

Deficits in stress-response systems are a characteristic of schizophrenia and psychosis spectrum illnesses, and recent evidence suggests that this impairment may be evident in those at clinical high-risk (CHR) for the development of a psychotic disorder. However, there is limited research specifically investigating biological and subjective stress reactivity in CHR individuals. In the present study, 38 CHR individuals and group of 38 control individuals participated in the Trier Social Stress Test (TSST), an experimentally induced psychosocial stressor. Changes in salivary cortisol and alpha amylase, as well as self-reported units of distress (SUDS), were evaluated. Interestingly, the TSST did not induce a change in cortisol levels in either group, though the CHR group did show higher overall cortisol levels throughout the TSST (pre-anticipation period through recovery period). However, indicative of an effective task manipulation, the TSST did illicit an increase in alpha amylase in both groups. CHR participants exhibited higher levels of subjective stress prior to the stressor compared to the control group and CHR SUDs did not significantly increase in response to the stressor. In contrast, the control group showed an increase in SUDS in response to the stressor. Notably, SUDS for the control group post task mirrored the levels CHR youth endorsed prior to the stressor. Taken together, these findings suggest that there may be a functional relationship between persistently elevated cortisol and chronic high levels of subjective distress in CHR individuals.

## Introduction

Psychotic disorders are extremely debilitating, chronic, and costly illnesses (1, 2); therefore, there is a current drive to identify reliable markers that permit early detection and treatment that may then significantly improve long-term disorder progression and outcomes. A promising strategy is to utilize markers that are conceptually relevant to the diathesis-stress model to identify adolescents and young adults who are at risk for developing psychosis spectrum disorders, also known as individuals at clinical high-risk (CHR) for psychosis (3–5). A neural diathesis-stress conceptualization is an established model used to understand pathogenic processes driving the etiology of psychosis spectrum disorders, such as schizophrenia (4, 6, 7). Specifically, the model predicts that an early vulnerability to normal hypothalamic-pituitary-adrenal (HPA) axis function, later interacts with normative and pathological neuromaturational processes and environmental stressors during the critical developmental period of adolescence. There is a growing and rich evidence base assessing specific stress systems that has largely focused on the HPA axis and basal cortisol levels in CHR individuals (8–10). However, very little of this work has examined biological and psychological domains of reactive stress. This is problematic given the complex interaction between biological stress systems and emerging psychopathology during adolescence, a complex developmental period of biological and psychosocial change (11).

The HPA axis is a dynamic pluripotent stress response system which contributes to the important circadian regulation of the body through a prominent daily rhythm of basal cortisol secretion (12). Thus, the HPA axis helps mediate the appropriate adaptive response to stress across the day, and dysfunction in one aspect of HPA axis operation has implications for overall HPA axis functional outcomes (13). Interestingly, there appears to be an inverse relationship between basal and reactive cortisol levels in schizophrenia patients, in which patients with schizophrenia display elevated basal levels (14), but a blunted response to stress (15, 16). Recent investigations observe similar elevated basal cortisol in youth who are at risk for psychosis (10, 17, 18), however, group differences are not always observed (4). Varying results suggest that basal cortisol levels alone might not be sufficient in identifying risk status.

Few studies have examined both hormonal and psychological responses to psychosocial stress in CHR individuals and results have been inconsistent. In a study examining psychological stress induced by the Montreal Imaging Stress Test (MIST), significantly greater salivary cortisol response to the stressor was found in the CHR group compared to the healthy controls (19). Alternatively, a blunted cortisol response was observed in a group of CHR individuals compared to healthy controls in response to the Trier Social Stress Test (TSST), and there were no significant group differences in global ratings of self-reported stress experience during the task (20). Heterogeneity in sample and methods across studies, as well as limited power, may have led to divergent findings. For example, risk samples ranged from sizes such as 12 (19) to 21 (20). Additionally, some studies use the Comprehensive Assessment for At Risk Mental States (CAARMS) to define risk (20), while others used the Structured Interview for Prodromal Symptoms [SIPS (19)].

Given that stress reactivity depends on the interplay of multiple neurophysiological systems (21, 22), there is a critical need for further evaluations that build on these studies by incorporating multiple measures of stress reactivity. Incorporating the sympathetic nervous system (SNS) offers a promising addition to further understanding stress reactivity and psychosis risk. While the SNS has traditionally not been incorporated into the neural diathesis-stress model of psychosis, the control and function of the HPA-axis, and SNS are interdependent and dysregulation in one system has implications for the other (23–25). The SNS speed of onset is rapid while the duration of its action is short lasting, as opposed to the HPA axis' slow speed of onset and long lasting duration of action (26, 27). The differential time-course of each system means that each provides unique and important information throughout the stress experience that can be overlooked if not studied in tandem (27, 28).

The present study is the first to examine group differences in salivary cortisol (HPA-axis), salivary alpha amylase (SNS), and subjective response to an acute lab stressor experimental paradigm in CHR adolescents and a control group of adolescents. Based on evidence indicating a blunted hormonal stress response in patients with schizophrenia (15), it was predicted that the risk group will exhibit blunted cortisol levels in response to the stressor relative to the control group. Further, given the parallel relationship between SNS response and subjective stress and evidence indicating that CHR individuals report higher levels of distress (20, 29), it is predicted that the CHR group will have higher alpha amylase levels and report elevated subjective stress in response to the social stressor compared to the control group.

## Methods

### Participants

Participants were recruited at the Adolescent Development and Preventative Treatment (ADAPT) research program and a total of 76 (38 CHR and 38 control) adolescents participated in the study. Participants were recruited by Craigslist, e-mail postings, newspaper ads, bus ads, and community professional referrals. Exclusion criteria included age <12 or >21 years of age, history of head injury, the presence of a neurological disorder, and lifetime substance dependence. The presence of an Axis I psychotic disorder was an exclusion criterion for CHR participants; however, other disorders were not exclusion criteria for CHR participants as comorbid Axis I disorders are typical of CHR individuals (30). Rates of current comorbid Axis I disorders in the CHR participants included 12 (32%) mood disorders, 14 (37%) anxiety disorders, and 1 (3%) ADHD. CHR individuals met criteria for a psychosis-risk syndrome including: (a) recent onset or escalation of moderate levels of attenuated positive symptoms (a score of 3–5 in the positive section of the Structured Interview for Psychosis-Risk Syndromes [SIPS P1-P5)], and/or (b) a decline in global functioning over the last 12 months accompanying the presence of schizotypal personality disorder (SPD), and/or (c) a decline in global functioning over the last 12 months accompanying the presence of a first-degree relative with a psychotic disorder such as schizophrenia (31). Meeting criteria for an Axis I disorder or the presence of a psychotic disorder in a first-degree relative were exclusionary criteria for the control group. The protocol and informed consent procedures were approved by the University of Colorado Institutional Review Board.

### Symptom Assessment

The Structured Interview for Psychosis-Risk Syndromes [SIPS (31)] was administered to diagnose a psychosis-risk syndrome. As noted, CHR participants in the present study met criteria for a psychosis-risk syndrome. The SIPS includes distinct categories of psychosis-risk symptom domains and a sum score for the positive symptoms section of the SIPS (P1–P5) is used as an indicator of symptomatology. The Structured Clinical Interview for Axis-I DSM-IV Disorders [SCID-IV (32)] was administered to rule out formal psychosis in CHR participants and an Axis I disorder in the control group. All interviewers had inter-rater reliabilities that exceeded Kappa >80.

### The Trier Social Stress Test

**Figure 1:**
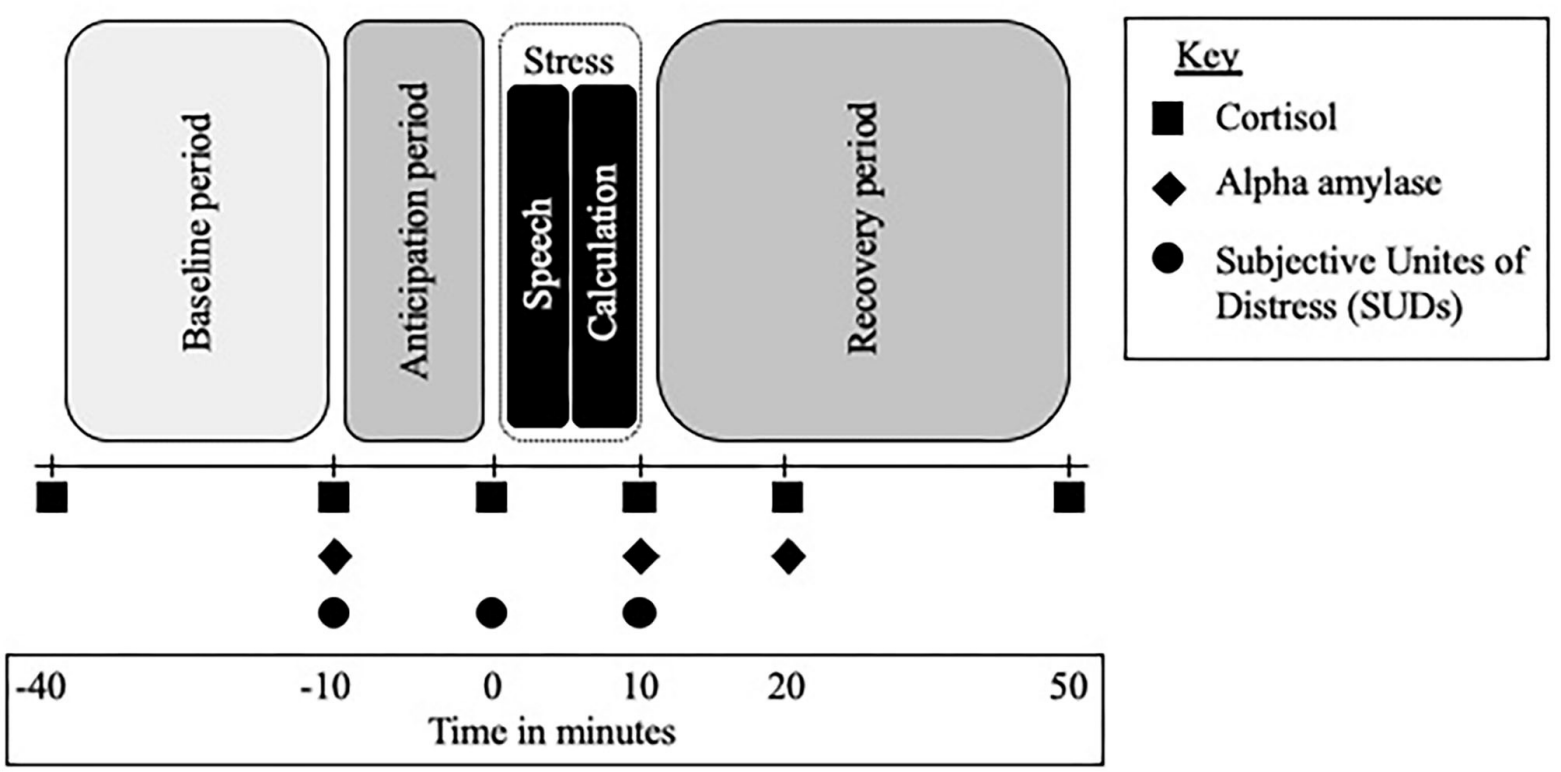
Trier Social Stress Test (TSST) timeline and procedure. TSST across time; minutes are in relation to the start of the stressor, anticipation refers to the time after the TSST instructions are given and the start of the stressor, the stressor lasts for 10 min and includes a 5-min speech and 5-min math challenge.

A modified version of the Trier Social Stress Test [TSST (33)] was used to measure participants' biological (HPA axis and SNS) and psychological (subjective report) response to the acute psychosocial stress. The TSST included a baseline period (40 min prior to task), an anticipation period (10 min), followed by a test period (10 min) during which participants gave a 5-min speech on a pre-assigned topic (convince the judges that you were falsely accused of shoplifting) and then performed a mental calculation task for 5 min, all in front of video camera and one research assistant (participants were also told that a panel of judges will be viewing their video to evaluate at another time), and finally a recovery period (40 min); see Figure 1. A timer-alarm was used to signal collection times and all participants participated in the TSST in the afternoon.

The TSST is a robust procedure that induces a moderate psychosocial stressor and evaluates its effects on physiological and psychological response (33). Modifications to the number of live viewers and the speech topic are often made to make the task appropriate for the study population. Studies that have utilized the TSST with patients with schizophrenia and CHR individuals often do not have any live observers in the room with the participant and have participants conduct the task in front of a one-way mirror and/or video camera (16, 20, 34). The current study modified the task to include one live observer, in addition to the video recording, and changed the speech topic to convincing a judge that the participant was falsely accused of shoplifting. This is the first CHR study to conduct the TSST with a research assistant in the room with the participant and the modifications were made with the goal of increasing the stressfulness of the task.

#### Biological Stress Reactivity

HPA-axis reactivity was assessed by examining salivary cortisol levels at 6 points during the TSST. Timepoints allowed for the baseline period, acute reaction to the stressor, and recovery period of the HPA-axis to be captured. SNS reactivity was assessed by examining alpha amylase at three timepoints: the start of the anticipation period, at the end of the of the stressor, and 10 min into the recovery period; see Figure 1. Salivary alpha amylase is a well-documented biomarker of stress related SNS changes (35) and previous studies have examined alpha amylase in response to the TSST in a range of populations (36).

#### Subjective Stress Reactivity

The psychosocial stress level was measured by the Subjective Units of Distress Scale (SUDS) (37); a self-rated 100-point scale (1 = *not stressed at all*, 100 = *extremely stressed*). Participants were asked to orally report their levels of stress at three time points: the start of the anticipation period, right before the start of the TSST, and at the end of the TSST; see Figure 1. The SUDS is traditionally used in the TSST to measure subjective stress and has been used with adolescents, young adults, and CHR individuals (38).

### Saliva Collection and Processing

Based on prior studies, saliva collection utilized a passive-drool method (39, 40). Participants were provided written and verbal dietary instructions to observe the evening before and the morning of sampling day. Because diet, activity, and medication affect cortisol, subjects were given explicit instructions and provided a log to detail these activities. On the evening before saliva sampling, participants were asked to avoid alcohol and caffeine after 6:00 P.M. On the morning of the assessment, they were asked to refrain from physical exercise and caffeinated beverages, to consume only grains, milk, juice or water, and to avoid over-the-counter medications. Subjects were questioned to confirm their compliance with the instructions. Saliva samples (75 μl) were stored in a −20°C freezer until time of assay. Salimetrics, LLC, College Park, PA was used to process and assay samples. Following gold standard procedures, samples were subjected to duplicate analyses and then averaged. Participants with missing samples, samples with an extremely low value (<0.007 μg/dL cortisol, 5.1 pg/ml alpha amylase; sensitivity cut-off value recommended by Salimetrics), and samples collected outside of the designated testing period were excluded from the study (cortisol for 2 control participants). Additionally, 8 CHR and 10 control individuals did not provide enough saliva for alpha amylase to be assayed confidently; therefore, alpha amylase levels were not available for the full sample.

### Statistical Approach

Analyses were completed using SPSS Statistics 24 software. Independent *t*-tests and chi-square tests were employed to examine differences between groups in respective continuous and categorical demographic variables such as age, sex, and parental education, along with total positive symptoms. To test for changes in cortisol levels between groups over time, a 2-factor mixed design ANCOVA (time × group) controlling for age was conducted and 2-factor mixed design ANOVAs (time × group) were conducted to examine changes in alpha amylase and SUDS by group over time. Consistent with previous work (17, 39, 41), cortisol statistics controlled for age due to the observed correlation between age and cortisol levels to avoid any possible effect of age (42). Greenhouse-Geisser and Huynh-Feldt corrected results were employed when sphericity assumptions were violated, and the epsilon estimate was used to determine type of correction. *Post-hoc t-*tests were employed to follow-up significant interactions to examine group differences at each timepoint. In the analysis of group differences in cortisol, the first cortisol sample collected (−40 min) was dropped from the analysis to exclude potential confounds (i.e., traveling to session, adjusting to lab setting), which is consistent with previous studies (20). Additionally, given 4 (10.5%) CHR participants were taking antipsychotic medications and 3 (7.9%) CHR participants and 2 (5.3%) control participants reported taking birth control, main analyses were run with and without these participants. Excluding participants taking these medications did not change the results; thus, all analyses reported include these participants.

## Results

### Demographic and Clinical Characteristics

As noted, a total of 76 (38 CHR and 38 control) adolescents participated in the study. There were no significant differences in age [*t*(62.09) = 1.14, *p* = 0.260], in years of education [*t*(73) = 0.01, *p* = 0.996], or sex [χ^2^(1) = 0.84, *p* = 0.358] between the CHR and control groups. As expected, the CHR group showed significantly more positive SIPS symptoms [*t*(39.88) = 16.11, *p* < 0.001] when compared with healthy controls. See Table 1 for a detailed report of self-reported demographic and clinical characteristics.

**Table 1:** Self-reported participant demographics & symptom characteristics.

**Group**	**CHR**	**Control**	**Statistic**	***p*-value**
**Gender**				
Female	16 (42%)	20 (53%)		
Male	22 (58%)	18 (47%)		
Total	38	38	χ^2^ (1) = 0.84	0.358
**Age**				
Mean years (SD)	18.87 (1.66)	18.29 (2.66)	*t*(62.09) = 1.14	0.26
**Education**				
Mean years (SD)	12.41 (1.98)	12.41 (2.52)	*t*(73) = 0.01	0.996
**Ethnicity**				
Hispanic	9 (24%)	10 (26%)		
Non-hispanic	29 (76%)	28 (74%)	χ^2^ (1) = 0.07	0.791
**Race**				
First Nations	0 (0%)	0 (0%)		
East Asian	1 (3%)	3 (8%)		
Southeast Asian	0 (0%)	1 (3%)		
Black	1 (3%)	1 (3%)		
Central/South American	8 (21%)	9 (24%)		
West/Central Asia & Middle East	1 (3%)	2 (5%)		
White	26 (68%)	21 (55%)		
Interracial	1 (3%)	1 (3%)	χ^2^ (6) = 2.92	0.818
**Parent education**				
Mean (SD)	16.92 (2.29)	16.18 (2.82)	*t*(73) = 1.24	0.22
**SIPS symptoms**				
Positive mean (SD)	12.42 (4.48)	0.21 (0.91)	*t*(39.88) = 16.11	<0.001
**Medication**				
Antipsychotics	4 (11%)	0 (0%)	χ^2^ (1)=4.22	0.04

*Positive symptoms reflect total sums from domains from the Structured Interview for Psychosis-Risk Syndromes (SIPS, P1–P5)*.

### Clinical Group Differences in Acute Stress Reactivity

**Figure 2:**
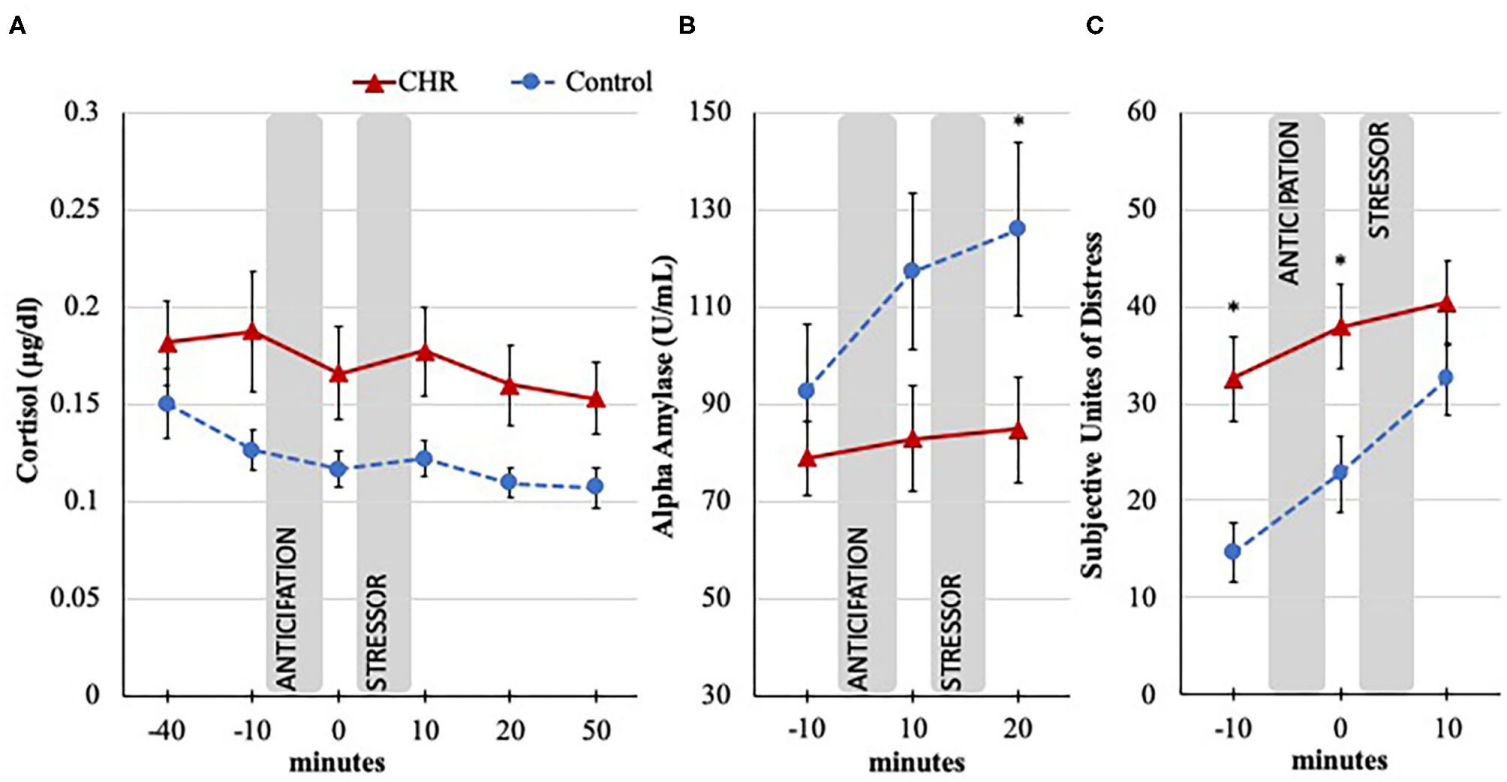
Cortisol, alpha amylase, and SUDS response to the TSST in CHR and control groups. Trier Social Stress Test (TSST), minutes are in relation to the start of the stressor, anticipation = time between instructions, and stressor; Error bars represent standard error of the mean. **(A)** Salivary cortisol response measured in μg/dl; −40 timepoint dropped from analysis to exclude potential confounds of travel; Omnibus effect significant (*p* < 0.05) for between group differences. **(B)** Salivary alpha amylase response measured in U/ml; Omnibus effect significant (*p* < 0.05) increase in both groups; *p < 0.05 for *post-hoc* group difference. **(C)** Subjective psychological response during the TSST measured with the Subjective Unites of Distress Scale (SUDS; 1 = *not stressed at all*, 100 = *extremely stressed);* Significant group by time interaction (*p* < 0.05); *p < 0.05 for *post-hoc* group difference.

In the examination of cortisol levels across the TSST, the group by time interaction and main effect of time were not significant [group × time: *F*(1.7, 121.13) = 0.22; *p* = 0.768; time effect: *F*(1.7, 121.13) = 0.60; *p* = 0.524; Greenhouse-Geisser correction]. These results suggest that the TSST stressor did not induce a significant change in cortisol in either group. However, the analysis revealed an overall main effect of group where cortisol levels during the TSST were significantly higher in the CHR participants compared to the control group [*F*(1, 71) = 3.97; *p* = 0.050; Greenhouse-Geisser correction], see Figure 2A.

Interestingly, while there was not a significant group by time interaction for alpha amylase [*F*(2, 112) = 2.18; *p* = 0.118], levels in both groups increased in response to the TSST as indicated in the main effect of time [*F*(2, 112) = 4.42; *p* = 0.014], see Figure 2B. In the absence of a cortisol effect, these results are important as they suggest that the TSST manipulation was in part effective in eliciting a response. It is also noteworthy that it appears that the CHR group's alpha amylase levels did not increase at the same rate as the control group, as evidenced by a weak trend-level main effect result for group, [*F*(1, 56) = 3.06; *p* = 0.086]. Exploratory *post-hoc* tests indicate that lower CHR's alpha amylase levels after the stressor (*p* = 0.052) appear to be driving the trend result. This pattern is supported by follow-up AUC_i_ analysis provided in the Supplementary Material.

In the examination of group difference across the TSST in subjective stress experience, results revealed a significant group by time interaction [*F*(1.86, 137.65) = 3.43; *p* = 0.038; time effect: *F*(1.86, 137.65) = 21.00; *p* < 0.001; group effect: *F*(1, 74) = 7.03; *p* = 0.010; Huynh-Feldt correction], see Figure 2C. Following the omnibus tests, *post-hoc* analyses revealed an interesting pattern where CHR individuals started and remained at an elevated level compared to controls before and after the anticipation period prior to the stressor (*p* = 0.001 and *p* = 0.011, respectively). However, by the time the stressor was over, the SUDS level of control group increased to a level that was comparable to the CHR level (*p* = 0.174). Results suggest that while the CHR group started the task with higher SUDS, the control participants experienced a larger increase in SUDS in response to the stressor compared to the CHR group.

## Discussion

The current study expands the small literature base examining biological and subjective stress reactivity to an acute laboratory stressor in CHR and control youth, and is the first study to include alpha amylase as a measure of SNS in this population. It was predicted that the CHR group would exhibit blunted cortisol levels in response to the stressor relative to the control group; however, group differences in cortisol reactivity to the TSST could not be evaluated as a significant change in cortisol after the TSST was not observed in either group. The results suggest that while the adapted version of the TSST used did induce a stress response as evident by the increase in alpha amylase levels (SNS response) and the SUDS, an HPA-axis response was not activated. Lack of HPA-axis activation has been observed in a previous psychosis risk study that used a different, but similar social stressor (43), as well as numerous other lab-based studies using the TSST with non-clinical samples (44). Interestingly, Pruessner et al. did observe an effect of cortisol and a blunted cortisol response in a CHR group with their use of an adapted version of the TSST (20). Such results are similar to findings observed in individuals with schizophrenia (15, 16), and suggest that HPA reactivity may be implicated during the prodromal phase of psychosis as well as during the chronic phase of illness. The lack of a cortisol response in the current study is more difficult to interpret. One possibility is that changing socio-cultural norms (e.g., time in front of cameras, few live observers, speech topic) resulted in a situation where the study's TSST failed to enact an HPA axis response, but continued to engage sympathetic adrenomedullary system arousal, as well as psychosocial subjective distress. Future studies that utilize a more stressful TSST experience and consider the impact of locus of control to perceived social stress (45) are needed to be able to better understand HPA-axis reactivity in CHR youth. However, it is notable that the CHR group in the current study exhibited significantly higher cortisol levels across the testing period, which is consistent with previous reports of elevated basal cortisol levels (10, 17, 18).

Salivary alpha amylase was included in the protocol as a second biological marker of stress reactivity (SNS). The SNS speed of onset is more rapid than the HPA response (presently measured by cortisol) and it was predicted that the CHR group will have higher alpha amylase response compared to the control group. Interestingly, an increase in alpha amylase levels was observed in both groups in response to the TSST (though the CHR group's increase was slightly smaller compared to the control group, at a weak trend level), which indicates that a physiological stress reaction was induced by the stressor. Given the lack of HPA-axis reactivity observed, the inclusion of salivary alpha amylase provides an important window into biological reactivity that would have been missed had it not been examined. The results suggest that the fast acting SNS response is a useful measure within this population. This is promising as studies examining other markers of the autonomic nervous system and SNS reactivity have reported mixed results among patients with schizophrenia and CHR individuals (46, 47). Interestingly, a blunted blood pressure in response to TSST has been observed in a group of CHR youth when compared to controls, though these youth exhibited similar increases in heart rate (20). These findings complement the trend result in the current study of the CHR participants possibly having lower overall alpha amylase levels across the TSST. Notably, heart rate, blood pressure, catecholamine levels, and salivary alpha amylase are all part of the SNS response, yet they measure different parts of the system and have different speeds of response (35), and this could account for varied results. While the available findings are preliminary and need to be interpreted with caution, they suggest that abnormalities in SNS reactivity may be present in CHR youth and salivary alpha amylase could potentially be a more sensitive measure of dysregulated biological stress reactivity than cortisol levels alone. It is also possible that the combination of a lower alpha amylase response with elevated cortisol may serve as a more sensitive marker of CHR than either alone; therefore, future longitudinal studies should specifically examine potential negative correlations between these two systems in CHR youth.

Contrary to the hypothesis that the CHR group would report elevated SUDS in response to the stressor compared to the control group, a pattern emerged showing that the CHR group experienced higher subjective stress at the first two timepoints prior to the stressor without a notable increase in response to the TSST. The control group did show the expected pattern of increase in response to the stressor. Notably, the CHR group's SUDS at baseline appeared similar to the control group's levels post stressor. This pattern provides a unique perspective of subjective stress experience over time and suggest that timing is important. One interpretation of these results is that the CHR in this sample came into the study feeling more stressed than the control group and the CHR subjective stress levels did not have the capacity to increase at the rate of the control group. Higher SUDS prior to the stressor in the current study is consistent with previous findings indicating that CHR individuals report elevated levels of perceived chronic stress (8, 29, 43). The lack of group differences in SUDS after the TSST is also consistent with previous findings in CHR populations when the overall stressfulness of a TSST was evaluated (20), and in response to a social stress criticism task (48). The combination of results suggests that individuals at risk for developing psychosis may chronically feel more stressed than their peers and their psychological stress levels do not react to reach higher levels in response to acute stressors.

Given the combination of the lack of SUDS reactivity and the trend result suggesting a weaker alpha amylase response in the CHR group, another interpretation to consider is that the CHR group may not have experienced the experimental stressor to be as stressful as the control group. Deficits in Theory of Mind (ToM), the ability to infer and understand others' mental states, have been observed in CHR groups (49). Thus, it is possible that the CHR individuals did not engage with the second part of the experimental stressor (defend self in a hypothetical shoplifting scenario) to the same degree as the control participants. This is interesting to consider given evidence of higher overall ratings of subject distress have been observed in a study where a psychosis risk group participated in a Virtual Reality environmental social stress task (38); a task that potentially requires less ToM. Future studies should continue to explore the possible impact of ToM on experimental stressors in CHR samples, especially when utilizing the TSST. Overall the SUDS results highlight the importance of measuring variables of interest overtime, not just at one time point or using an overall rating when examining stress reactivity.

Taken together, the results of the study replicate previous observations of higher overall cortisol levels in CHR individuals and introduces the consideration of the impact of persistently high subjective stress in the dysregulation of acute stress reactivity in psychosis risk. It is possible that chronically high levels of subjective stress could lead to a decreased sensitivity and subjective reactivity to stressors and cause dysregulation to biological stress systems, such as the alteration of the negative feedback of the HPA-axis believed to occur in patients with schizophrenia (50). Interestingly, there is some evidence to suggest that while individuals at elevated risk for psychosis have similar affective response after a social stressor compared to low risk controls, they may have difficulty regulating their physiological response (as measured through heart rate) (48). Future longitudinal studies are needed and should be specifically designed to account for the role of culture and the vast hormonal, motivational, and social changes that take place during development, as these factors are likely to influence vulnerabilities in stress response and symptom progression (51, 52). Further, in light of recent evidence regarding sex differences observed in schizophrenia patients and youth identified as being at risk for developing psychosis (4, 53, 54), as well as an update to the neural diathesis-stress model of schizophrenia that specifically highlights sex differences as an important future direction (4), future studies with adequate power are needed to evaluate potential sex effects. Such future work can deepen the conceptualization of the diathesis stress model and offer useful intervention targets, both of which increase the potential to impact the lives of CHR young adults and their families.

The findings from the present study should be interpreted in the context of its limitations. First, while the overall sample size was larger than previous studies that examine stress reactivity among CHR individuals (19, 20, 43), the alpha amylase analysis was conducted in a subset of the overall sample size and resulting lack of power could account for the trend results observed. This is the first study to include alpha amylase as a measure of SNS activity in a CHR sample and studies with greater power that utilize multiple levels of analysis should be conducted before any definitive conclusions are made. Relatedly, information about menstrual cycle and wake-up time on the day of the TSST was not collected; therefore, the study could not control for possible confounds related to cycle (55) or differences in diurnal variations in cortisol and alpha amylase. It is important for future studies to collect this data and examine possible confounds. It is also recommended that future studies use a broader age range than the current study (12–21 years old) to account for lifestyle changes that occur during the transition from adolescents to young adulthood that may impact stress reactivity (e.g., high school students and college students sleep and activity schedules tend to differ). Finally, it was not possible to evaluate one of the main hypotheses about cortisol reactivity due to the lack of a cortisol response to the adapted version of the TSST. The study used a modified version of the TSST and modifications were similar to previous studies; however, it is possible that the results may have been impacted by the modifications. It is recommended that future studies that use an experimental stressor, especially the TSST, carefully decide on appropriate procedures that are specific to the demographics, values, and cognitive abilities of the study population and consider time of day.

## Data Availability Statement

The raw data supporting the conclusions of this article will be made available by the authors, without undue reservation.

## Ethics Statement

The studies involving human participants were reviewed and approved by University of Colorado Institutional Review Board. Written informed consent to participate in this study was provided by the participants' legal guardian/next of kin.

## Author Contributions

Material preparation and data collection were conducted by EC and VM. Data analysis was performed by EC with feedback and guidance on analysis plan and interpretation by VM and RS. VM and EC provided funding for the project. All authors contributed to the manuscript, study conception, and design.

## Conflict of Interest

The authors declare that the research was conducted in the absence of any commercial or financial relationships that could be construed as a potential conflict of interest.
