# Supplementary material for: Acute Physiological and Psychological Stress Response in Youth at Clinical High-Risk for Psychosis
Source: Front Psychiatry. 2021 Feb 19;12:641762. doi: 10.3389/fpsyt.2021.641762 (PMC7933586; doi:10.3389/fpsyt.2021.641762)
Supplement: Supplementary file 1 [file Data_Sheet_1.PDF]

Supplementary Material

1 Supplementary Data

Follow-up analysis was conducted to explore the trend-level main effect observed in the alpha amylase analysis. Consistent with previous work (1), the area under the curve with respect to increase (AUC_i) was calculated to obtain a single value of alpha amylase for each group across the three time points (2). The AUCi represents the amount of change that occurs across the testing period. An independent t-test was used to examine group differences. Results revealed higher AUC_i in the control group compared to the CHR group at a trend level of significance [$t(59)=-1.75$, $p=0.086$].

2 Supplementary references

1. Pruessner M, Béchard-Evans L, Boekestyn L, Iyer SN, Pruessner JC, Malla AK. Attenuated cortisol response to acute psychosocial stress in individuals at ultra-high risk for psychosis. *Schizophr Res* (2013) **146**:79–86. doi:10.1016/j.schres.2013.02.019

2. Pruessner JC, Kirschbaum C, Meinlschmid G, Hellhammer DH. Two formulas for computation of the area under the curve represent measures of total hormone concentration versus time-dependent change. *Psychoneuroendocrinology* (2003) **28**:916–931. doi:10.1016/S0306-4530(02)00108-7
